# LINC00514 facilitates cell proliferation, migration, invasion, and epithelial-mesenchymal transition in non-small cell lung cancer by acting on the Wnt/β-catenin signaling pathway

**DOI:** 10.1080/21655979.2022.2084246

**Published:** 2022-06-02

**Authors:** Yuanzhe Zhu, Huala Wu, Xi Yang, Zhijuan Xiong, Tiantian Zhao, Xin Gan

**Affiliations:** a Department of Respiratory and Critical Care Medicine, The First Affiliated Hospital of Nanchang University, Nanchang, People’s Republic of China; b Jiangxi Institute of Respiratory Disease, The First Affiliated Hospital of Nanchang University, Nanchang, People’s Republic of China; c Department of Oncology, The First Affiliated Hospital of Nanchang University, Nanchang, People’s Republic of China

**Keywords:** LINC00514, NSCLC, proliferation, migration, invasion, EMT, wnt/β-catenin

## Abstract

The long non-coding RNA (lncRNA) LINC00514 was identified to play an essential oncogenic function in different human cancers, but its effects in non-small cell lung cancer (NSCLC) are yet to be elucidated. In this study, we evaluated the function of LINC00514 in NSCLC. LINC00514 expression and prognosis in NSCLC were analyzed using qRT-PCR and online bioinformatic tools. The bioeffects of LINC0514 in NSCLC cells were examined using cell counting kit-8, colony formation, and transwell assays. Western blotting was used to measure the expression of the target proteins. The LINC00514 regulation of the Wnt/β-catenin signaling pathway was assessed using a specific agonist (LiCl) and luciferase reporter assay. We found that LINC00514 expression was elevated in NSCLC cells and clinical samples and that increased LINC00514 expression predicted poorer patient prognosis. Silencing LINC00514 suppresses proliferation, migration, and invasion of NSCLC cells. Downregulation of LINC00514 inhibited Wnt/β-catenin signaling and epithelial-mesenchymal transition (EMT). Moreover, suppression of the biological phenotypes of NSCLC cells induced by LINC00514 gene silencing was restored after LiCl treatment. Finally, we found that silencing LINC00514 attenuated the growth of xenograft tumors in vivo. Altogether, this study provides the latest convincing evidence that LINC00514 facilitates the malignant biological behavior of NSCLC cells through activation of the Wnt/β-catenin pathway, which might offer a beneficial approach for the treatment of NSCLC.

## Graphical abstract

**Figure:**
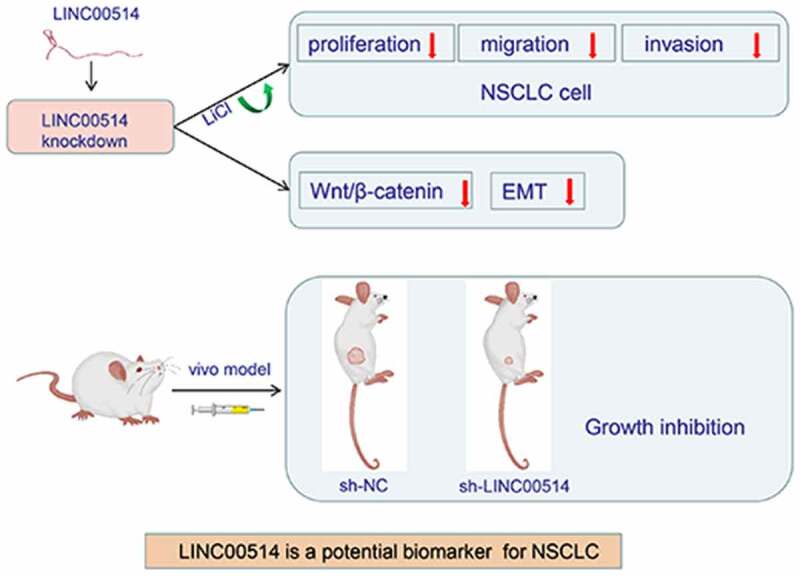

## Highlights

- Higher LINC00514 expression was observed in NSCLC tissues and cells.
- Increased LINC00514 expression predicted lower patient survival rates.
- Silencing LINC00514 suppressed proliferation, migration, invasion, and EMT of NSCLC cells.
- LINC00514 exerts oncogenic effects through the Wnt/β-catenin signaling pathway.

## Introduction

Based on the latest global cancer data released in 2020, lung cancer morbidity and mortality rates are as high as 11.4% and 18%, and remain the primary causes of the death in people with cancer [1]. As the dominant pathological type of lung cancer, non-small cell lung cancer (NSCLC) accounts for more than 80% of lung cancer cases [2]. Improvements in traditional chemotherapy regimens and precision molecular treatments, such as immune checkpoint inhibitors, have significantly improved outcomes in patients with NSCLC; however, only 26% have survived for more than five years after diagnosis [3]. Hence, there is a great need to uncover the underlying mechanisms of NSCLC pathogenesis in order to identify relevant markers for early diagnosis, prevention, and treatment.

RNA involvement in gene modulation is prevalent in organogenesis and inheritance in higher organisms. In humans, over 90% of the transcriptional output is non-coding RNA, among which long non-coding RNA (lncRNA) refers to RNAs with transcriptional lengths between 200nt and 110kb. With the implementation of the ENCODE project, nearly 30,000 distinct species of lncRNAs have been identified in the human genome [4]. Ample evidence has demonstrated that lncRNAs play a pivotal role in the etiopathogenesis of many human disorders such as systemic lupus erythematosus, type 2 diabetes [5], hereditary diseases [6], and cancer. For example, lncRNAs guide phenotypic conversion in melanoma, lead to metastasis, influence patient response to immunotherapy, and trigger an adaptive response to therapy and acquisition of drug-resistant phenotypes [7]. Conversely, LINC00244 acts as a cancer suppressor gene to inhibit programmed cell death 1 ligand 1 (PD-L1) expression and effectively attenuate the aggressiveness of hepatocellular carcinoma [8].

Emerging investigations have confirmed the involvement of the Wnt/β-catenin signaling pathway in promoting the proliferation and invasion of NSCLC cells, while the activation of Wnt signaling in the tumor microenvironment plays an important role in assisting cell epithelial-mesenchymal transition (EMT) [9,10]. EMT can generate a range of cell morphologies that are intermediate between epithelial and mesenchymal cell characteristics, some of which show increased motility and disruption of cellular integrity that promotes the aggressiveness of cancer cells [11]. In recent years, as research on lncRNAs has intensified, studies have confirmed that lncRNAs contribute to this highly dynamic process by regulating the expression of EMT-related hallmarks. According to Huang et al., lncRNA FAM83A-AS1 regulates EMT in lung adenocarcinoma cells and promotes cancer cell invasion and metastasis [12].

As a functionally undefined lncRNA, LINC00514 has attracted the attention of many researchers. Han et al. reported that LINC000514 upregulates oncogene rap1b, thereby promoting pancreatic cancer development [13]. Another study demonstrated that LINC00514 in the nucleus of papillary thyroid cancer cells promotes tumor progression by elevating CD23 expression [14]. Similar trends have been reported in tumors, such as cervical cancer [15], triple-negative breast cancer [16,17], osteosarcoma [18], and esophageal cancer [19], but not in NSCLC.

We discovered that LINC00514 is highly expressed in NSCLC cell lines by searching through the Chinese National Center for Biological Information database (LncExpDB) [20]. Therefore, the present study aimed to explore the function and mechanism of action of LINC00514 in NSCLC. Specifically, we assessed the effect of LINC00514 on NSCLC growth, invasion, migration, and EMT. We confirmed that the Wnt/β-catenin signaling pathway may be involved in promoting tumor progression; thus, the goal of this study was to identify a potential biomarker for the precise treatment of NSCLC.

## Method

### Patient tissue samples

NSCLC tumors and corresponding normal samples were obtained from the First Affiliated Hospital of Nanchang University and were preserved in liquid nitrogen. The selected patients were all diagnosed with NSCLC for the first time and had not received radiotherapy or chemotherapy before sample extraction. Patient characteristics and relevant clinical information were obtained from the treatment records. This study was approved by the ethics committee of the First Affiliated Hospital of Nanchang University.

### Cell lines

The human bronchial epithelial (HBE) cells and NSCLC cell lines (H358, H1299, A549) were purchased from the Cell Bank of the Chinese Academy of Science. Cell lines were identified at the time of purchase to determine whether they were morphologically normal and free of viral and mycoplasma contamination. Cells were cultured in DMEM/RPMI-1640 medium supplemented with 10% fetal bovine serum (FBS; Yeasen Biotechnology, China) and maintained in a thermostatic incubator with 5% CO2 at 37°C. NSCLC cells were treated with the agonist lithium chloride (LiCl; 20 mM) to activate Wnt signaling.

### Short hairpin RNA (shRNA) knockdown

Short hairpin RNAs (shRNA; sh-LINC00514 and control sh-NC) were purchased from RiboBio (Guangzhou, China). According to the instruction manual, shRNA reagents were transfected into H1299/A549 cells using the Lipofectamine 3000 Transfection Kit (Invitrogen) [21]. qRT-PCR assays confirmed the knockdown effects of sh-LINC00514. We selected the most efficient shRNA to knock out, and the cells were transfected and incubated in medium containing 2 µg/mL puromycin to screen out positive colonies stably expressing shRNA.

### Quantitative real-time PCR assay

Total RNA was extracted from the samples using TRIZOL reagent (Invitrogen, USA) and reverse transcribed to cDNA using PrimeScript Reverse Transcriptase (Takara, Japan). SYBR green master mix (Vazyme) was used for PCR. The gene expression results were normalized to GAPDH, and relative quantification was calculated using the 2^−ΔΔCt^ method [8]. The specific primers used for analysis were: LINC00514 forward, 5’-GCTCAACATCTCACTTCTCCCAC-3’ and reverse, 5’-CCTTCAGTGTCTGGGAAAGAGAG-3’; GAPDH forward, 5’-GAGAAGGCTGGGGCTCATTT-3’ and reverse, 5’-AGTGATGGCATGGACTGTGG-3’.

### Western-blot assay

Total proteins were extracted from cultured cells and clinical samples using RIPA lysis buffer and quantified using a BCA Protein Quantification Kit. Samples were separated by 10% SDS-PAGE and transferred onto polyvinylidene difluoride (PVDF) membranes. The membrane was blocked with Tris-buffered saline with Tween-20 (TBST) containing 5% nonfat milk for 1 h and incubated with the corresponding primary antibody at 4°C overnight. After rinsing the membrane three times with TBST, it was incubated with species-specific horseradish peroxidase-labeled secondary antibody (1:5000) for 1.5 h at room temperature [12]. Primary antibodies against β-actin mouse mAb (1:5000; 23,660-1-AP) were obtained from Proteintech (Wuhan, China), while the antibodies against β-catenin rabbit mAb (1:1000; D10A8), E-cadherin rabbit mAb (1:1000; 24E10), N-cadherin rabbit mAb (1:1000; D4R1H), and cyclin D1 rabbit mAb (1:1000; E3P5S) were purchased from Cell Signaling (Danvers, USA). Antibodies were diluted according to the manufacturer’s recommendations.

### Cell proliferation assay

To assess the viability of NSCLC cells, we conducted a cell counting kit-8 (CCK8) assay [22]. After transfection, A549/H1299 cells were seeded in 96-well plates and incubated for 0, 24, 48, and 72 h, followed by the addition of 10 ul CCK8 reagent and incubated for 2 h in a constant temperature incubator. Subsequently, optical density was measured at 450 nm using an enzyme marker (Thermo Fisher, USA).

### Colony formation assay

After transfection, 1000 cells were seeded in each 6-well plate and incubated for 12 days. After washing three times with phosphate-buffered saline (PBS), the colonies were fixed using paraformaldehyde fixative and counted after crystal violet staining.

### Migration and invasion assays

Transwell chambers (Corning, Tewksbury, USA) with an 8 µm pore size were used. Approximately 1 × 10^5^ transfected cells were seeded in the upper chamber, whereas the medium in the bottom section was supplemented with 10% FBS. After incubation for 24 h, the cells that migrated to the lower chamber were stained with crystal violet and imaged under an EVOS FL microscope (Thermo Fisher, USA). Consistent with the migration assay protocol, the invasion assay was performed with a layer of Matrigel (Corning) covering the upper chamber side of the polycarbonate membrane [23].

### Luciferase reporter assay

TCF/LEF1-Luc luciferase reporter plasmids (Genomeditech, Shanghai, China) were transfected into LINC00514 knockdown H1299/A549 cells or negative control cells using Lipofectamine 3000 and incubated for 48 h. TCF/LEF-1 activity was assayed using a luciferase system (Promega, USA) [24]. Luciferase activity was normalized to the Renilla luciferase activity.

### In vivo xenograft model

Four to six weeks female nude mice were obtained from the National Laboratory Animal Center (Beijing, China). A total of 2 × 10^6^ A549 transfected cells were trypsinized and suspended in 100 μl PBS. Then, cell suspensions were inserted into the right axillary region of the mice (3 per group). The size of the xenograft tumors was recorded weekly using the following formula: tumor volume (mm^3^) = (length x width^2^) /2. Mice were euthanized after four weeks, and tumor tissues were excised, weighed, and photographed [13]. Mice were manipulated and housed according to the protocol provided by the Medical Animal Center of Nanchang University.

### Statistical analysis

SPSS 21.0 (IBM, NY, USA) and GraphPad Prism 8.3.0 (GraphPad Software, San Diego, USA) was employed for data analysis and presentation. Data were expressed as mean ± standard deviation, and statistical differences between the two groups were analyzed using Student’s t-test. For comparison among multiple groups, one-way ANOVA test was utilized followed by Post-Hoc Test. The data came from at least three independent experiments. The level of significance for all tests was set at P-value <0.05.

## Result

We used bioinformatics tools to predict the expression profile of LINC00514 in NSCLC tissues and cell lines, and conducted an experimental validation. Next, LINC00514 loss-of-function assays were performed to determine its roles in NSCLC cell proliferation, invasion, metastasis, EMT, and Wnt/β-catenin signaling. The rescue assay demonstrated that LINC00514 promotes NSCLC progression through the activation of Wnt signaling, and in vivo experiments further verified our hypothesis.

### LINC00514 was highly expressed in NSCLC and related to poorer prognosis

**Figure 1.**
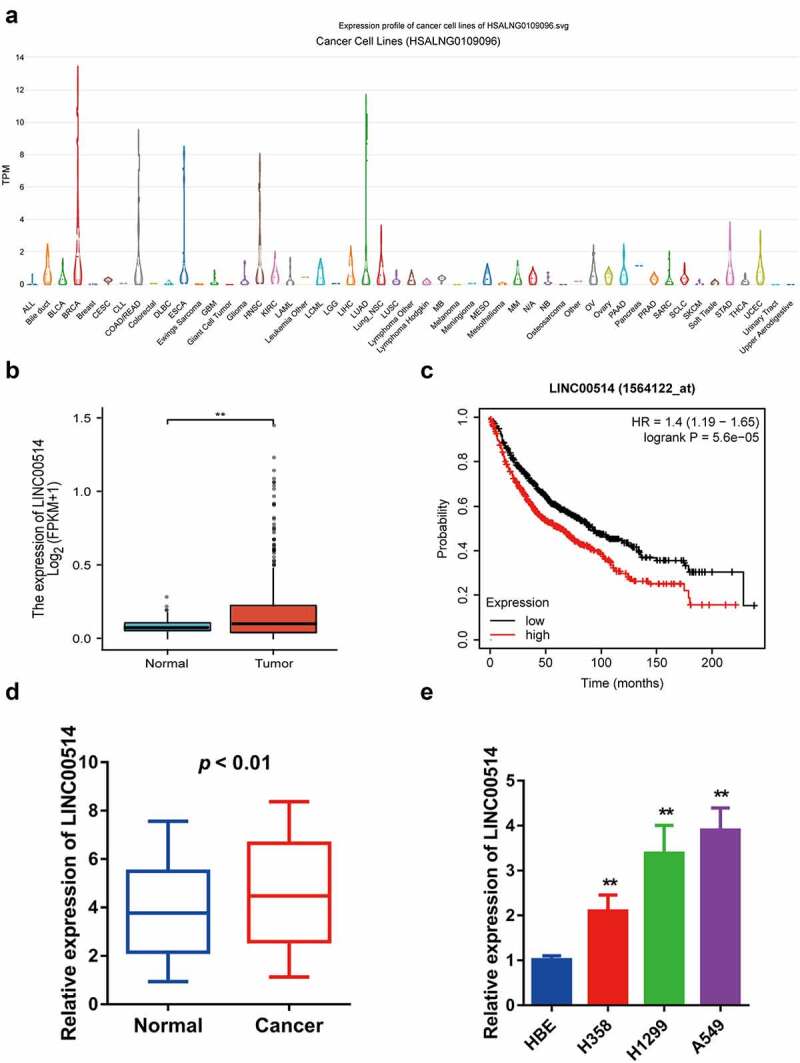
LINC00514 was upregulated in NSCLC and associated with a poor prognosis. (a) According to the LncExpDB database, LINC00514 was highly expressed in NSCLC lines, especially lung adenocarcinoma. (b) LINC00514 was significantly higher in NSCLC tumors (n = 535) versus normal tissues (n = 59) by analyzing the TCGA database. (c) Online Kaplan-Meier analysis assessed the association between LINC00514 expression level and overall survival in NSCLC patients (log-rank p 5.6e-5; HR 1.4; 95%CI 1.19–1.65; n = 1144). (d) qRT-PCR analysis showed increased expression of LINC00514 in NSCLC tumors versus adjacent normal tissues (n = 30). (e) LINC00514 was higher expressed in NSCLC cells. Three independent experiments were performed. Data are presented as the mean ± SD. LUAD, lung adenocarcinoma; qRT-PCR, quantitative real-time polymerase chain reaction; SD, standard deviation; NC, negative control; *p < 0.05, **p < 0.01, compared with normal tissues and HBE.

The LncExpDB database showed that LINC00514 was highly expressed in NSCLC cell lines, especially in lung adenocarcinoma (Figure 1(a)). We identified that LINC00514 expression was significantly higher in NSCLC tumors than in normal tissues by analyzing the TCGA database (Figure 1(b)). In addition, we evaluated the value of LINC00514 as a predictive biomarker for lung cancer using an online survival prediction tool [25], and the results suggested that high levels of LINC00514 were significantly correlated with poor overall survival (Figure 1(c)). Our qRT-PCR assay validated that LINC00514 was more highly expressed in NSCLC tumor tissues than in the adjacent normal tissues (Figure 1(d)). Likewise, LINC00514 levels were significantly elevated in NSCLC cell lines compared to those in HBE cells (Figure 1(e)).

### Downregulation of LINC00514 inhibited the malignant behaviors of NSCLC cells in vitro

**Figure 2.**
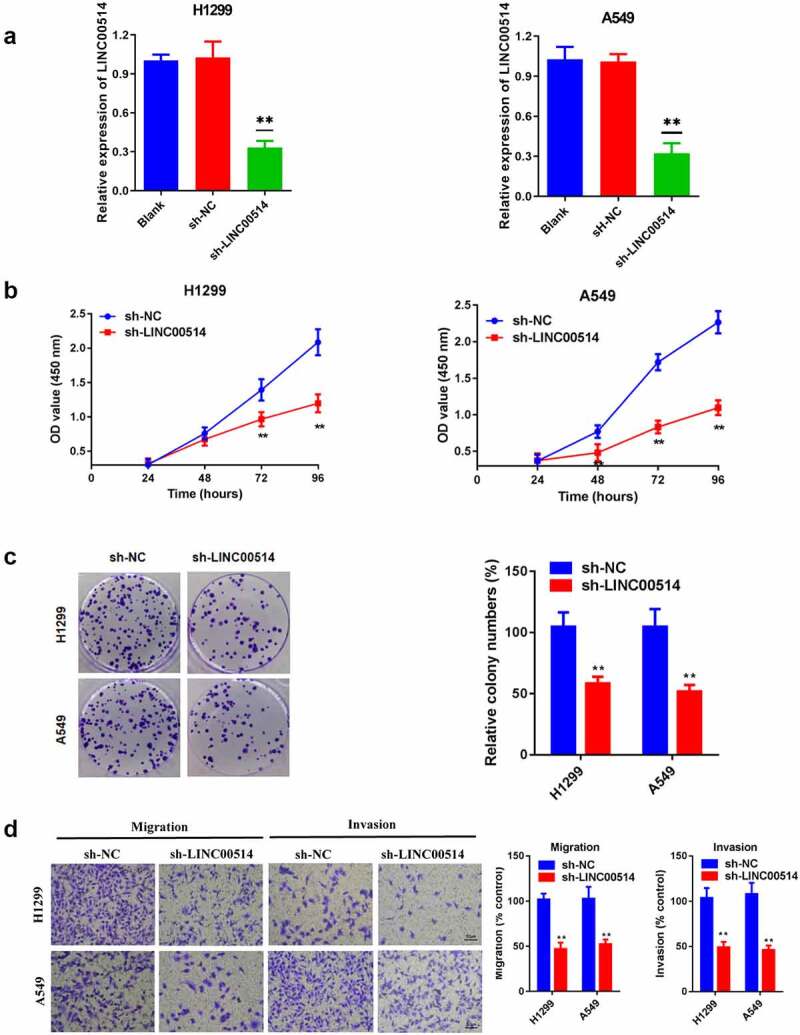
Silencing LINC00514 inhibited the proliferation, migration, and invasion of NSCLC cells in vitro. (a) Reduced expression of LINC00514 after transfection of H1299/A549 cells. (b) CCK-8 assay showed that suppression of LINC00514 restrained the cell proliferation of H1299/A549. (c) Colony formation assay indicated that cell proliferation capacity was reduced in LINC00514 knockdown H1299/A549 cells. (d) Transwell assay indicated that LINC00514 silencing inhibited the migration and invasion of H1299/A549 cells. Data are presented as the mean ± SD. CCK-8, cell counting kit-8; **p < 0.01, compared with sh-NC group, N = 3.

To further investigate the bioeffects of LINC00514 in NSCLC, we selected cell lines with high LINC00514 expression levels for knockdown. The qRT-PCR results indicated that transfection with sh-LINC00514 markedly decreased the expression of LINC00514 in A549 and H1299 cells (Figure 2(a)). Figure 2(b) shows the results of the CCK-8 assay, wherein the suppression of LINC00514 significantly restrained the capacity of A549 and H1299 cells to proliferate. Furthermore, the colony-forming ability of the cancer cells was impaired after LINC00514 knockdown (Figure 2(c)). We observed remarkably retarded migration and invasion of cells silenced by LINC00514 expression (Figure 2(d)). In summary, LINC00514 facilitated the proliferation, migration, and invasiveness abilities of cells.

### LINC00514 regulated EMT and Wnt/β-Catenin signaling pathway in NSCLC cells

**Figure 3.**
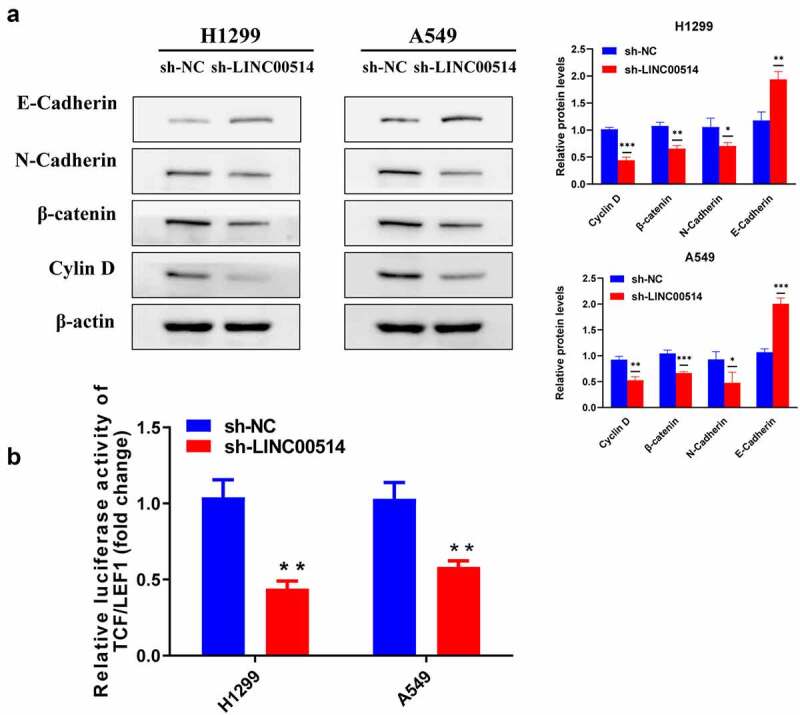
LINC00514 accounted for regulating EMT and Wnt/β-catenin signaling pathway in NSCLC cells. (a) Western-blot showed knockdown of LIN00514 affected EMT and Wnt/β-catenin signaling-related proteins. (b) TCF/LEF1 luciferase activity was suppressed by LINC00514 silencing. Data are presented as the mean ± SD. *p < 0.05, **p < 0.01, ***p < 0.001. compared with sh-NC group, N = 3.

Cancer cells have higher migratory and invasive capacities after EMT. Combined with the analysis of the above phenotypes, we speculated that the effect of LINC00514 on lung cancer may be related to the promotion of EMT. We then detected EMT-related proteins. As shown in Figure 3(a), after sh-LINC00514 transfection of H1299/A549 cells, E-cadherin expression was elevated, while N-cadherin was reduced. Since Wnt signaling is a common upstream signal of EMT, we detected changes in key point β-catenin and downstream protein cyclin D1 after transfection, both of which were significantly decreased in NSCLC cells after silencing of LINC00514. Likewise, the luciferase reporter assay indicated that TCF/LEF1 activity was suppressed by LINC00514 silencing, further indicating that LINC00514 knockdown suppressed Wnt signaling in NSCLC cells (Figure 3(b)).

### Downregulation of LINC00514 attenuated malignant activities in NSCLC cells by modulating the Wnt/β-Catenin signaling pathway

**Figure 4.**
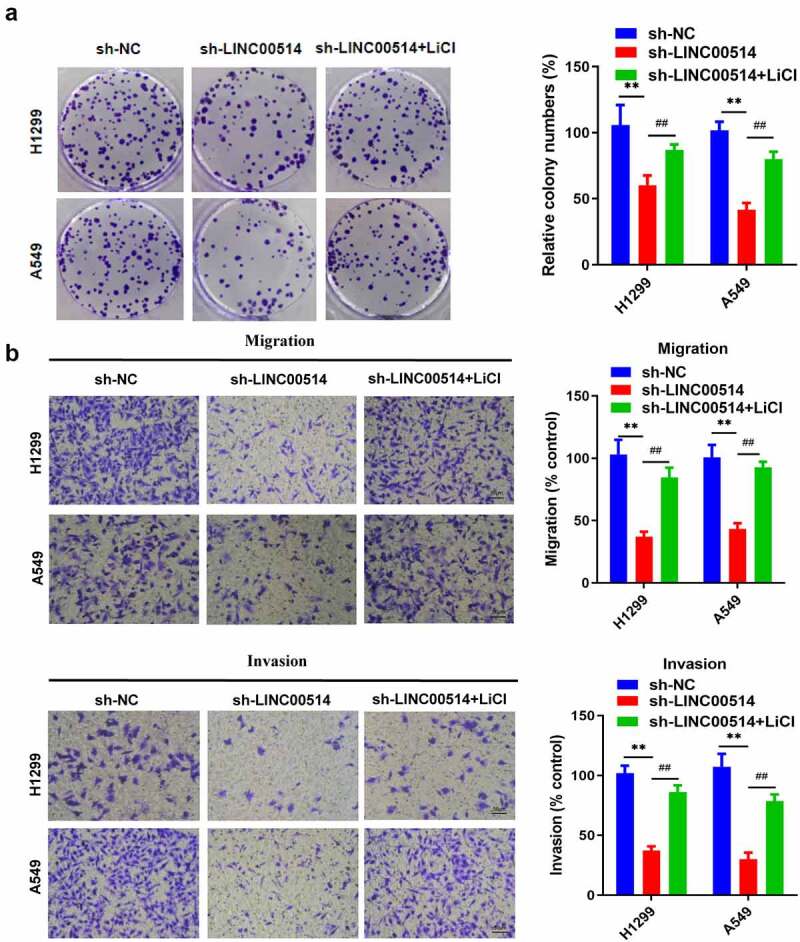
LINC00514 expression led to NSCLC cancer progression by modulating the Wnt/β-Catenin signaling. (a) Colony formation assay showed that silencing LINC00514 resulted in decreased cell proliferation, restored by the Wnt agonist LiCl. (b) Transwell assay further showed that LiCl could recover migration and invasion rate of LINC00514 knockdown H1299/A549 cells. LiCl, Lithium chloride; Data are presented as the mean ± SD. **p < 0.01, compared with sh-NC group; ##p < 0.01, compared with sh-LINC00514 group, N = 3.

To further prove the association between LINC00514 and Wnt signaling, LINC00514-silenced NSCLC cells were cultivated with LiCl, a specific agonist of Wnt signaling. Subsequent colony formation experiments demonstrated that LiCl significantly increased the proliferative capacity of LINC00514-silenced H1299 and A549 cells (Figure 4(a)). Additionally, LINC00514-silenced cells recovered their migration and invasion abilities in the presence of LiCl (Figure 4(b)). Our results confirmed that high LINC00514 expression leads to NSCLC cancer progression in a Wnt/β-catenin-dependent manner.

### Silencing of LINC00514 attenuated NSCLC tumor growth

**Figure 5.**
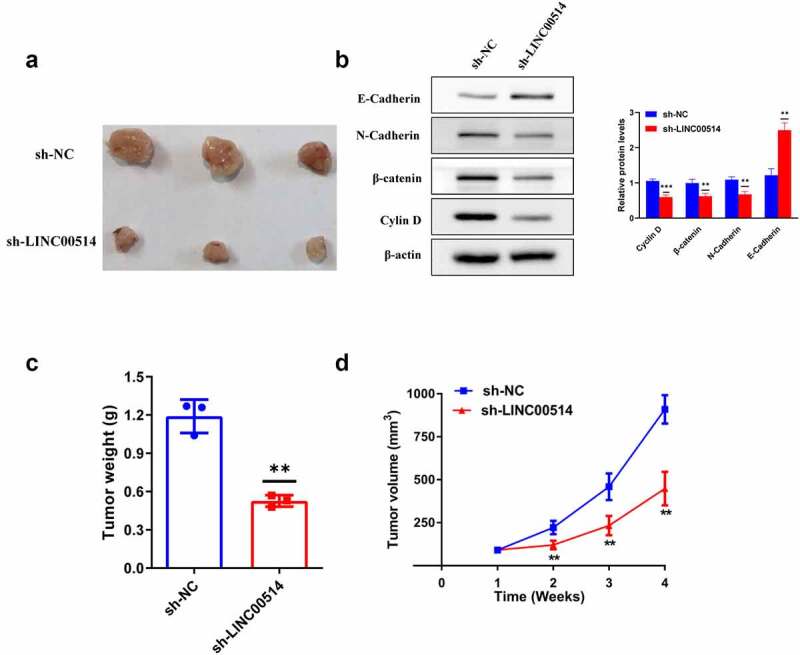
Silencing of LINC00514 attenuated NSCLC tumor growth in vivo. (a) The images of subcutaneous tumors were obtained on Day28. (b) Western-blot showed that knockdown of LINC00514 affected the expression of EMT and Wnt/β-catenin signaling-related proteins in vivo. (C/D) The tumor volumes and weights of the sh-LINC00514/sh-NC group were quantified. Data are presented as the mean ± SD. **p < 0.01, ***p < 0.001. compared with sh-NC group, N = 3.

Tumorigenic assays in nude mice were used to assess the effects of LINC00514 on NSCLC in vivo. A549 cells transfected with sh-LINC00514 or sh-NC were injected into the nude mice. Tumor volumes were calculated weekly after injection, and tumors were obtained for weighing after euthanasia of the mice after four weeks. The results showed that tumor xenograft growth was attenuated in the sh-LINC00514 group (Figure 5(a, c, d)). In addition, we found that LINC00514 silencing caused an increase in E-cadherin expression and a decrease in N-cadherin, β-catenin, and cyclin D1 expression in xenograft tumors (Figure 5(b)). Taken together, these results demonstrated that LINC00514 plays an important cancer-promoting role in NSCLC in vivo.

## Discussion

As a malignancy with a high incidence and lethality worldwide, NSCLC poses a major threat to human health. Searching for its pathogenesis and effective therapeutic targets is in great need but also faced with many challenges. While ncRNAs were considered irrelevant in the transcription of genes when first discovered, emerging evidence suggests that they serve a vital function in organogenesis and human disease genesis [26]. Several studies have shown that mutations and dysregulation of some non-coding RNAs, especially lncRNAs, appear to influence the development of cancer, including NSCLC [27]. For example, highly expressed lncRNA MELTF-AS1 has been demonstrated to be associated with poorer survival in NSCLC patients [23]. The lncRNA CASC9 inhibits the oncogenic molecule DUSP1 by enhancing EZH2 expression, which in turn increases resistance to gefitinib in NSCLC [28]. LncRNA-XIST promotes NSCLC progression by inhibiting reactive oxygen species-mediated cellular scorch death [29]. Recent studies have revealed that LINC00514 also plays a vital role in the carcinogenesis of many malignancies, but its underlying role in NSCLC prognosis is still unknown.

In this study, we found that LINC00514 expression was remarkably increased in NSCLC tumor tissues and cell samples compared to that in non-tumor samples. Elevated LINC000514 expression predicted lower survival rates in patients with NSCLC. Furthermore, our study confirmed that LINC00514 enhanced the proliferative activity of NSCLC cells both in vivo and in vitro. The proliferation, migration, and invasion of NSCLC cells were markedly suppressed by silencing LINC00514. Invasion and metastasis, which are the most basic features of malignant tumors, are the major causes of the shorter life expectancy of patients [30]. These results indicated that LINC00514 may be important in the development and prognosis of NSCLC.

As a highly conserved evolutionary pathway, the Wnt/β-catenin signaling pathway has been extensively described in the pathogenesis of diseases [31]. Excessive Wnt signaling contributes to the generation and retention of cancer stem cells (CSCs), and plays a vital role in initiating malignancies in the colorectal, liver, breast, lung, and hematopoietic systems [32]. For instance, TM4SF1 activates Wnt signaling and promotes the expression of SOX2 to maintain stem cell properties and EMT in colorectal cancer, facilitating metastasis and recurrence [33]. TFAP4 activates Wnt signaling in vivo and in vitro by directly binding to the DVL1 and LEF1 promoters to enhance hepatocarcinoma cell tumorigenicity [34]. In canonical Wnt/β-catenin signaling, the presence of Wnt ligands suppresses the ubiquitinated degradation of β-catenin proteins, and the accumulated β-catenin proteins act as TCF/LEF family transcription factors co-stimulating the activation of target genes downstream of the signal to induce subsequent cellular responses [35]. In our study, LIN00514 knockdown decreased the expression of catenin and cyclin D1 proteins in NSCLC cells. Cyclin D1, a cell cycle protein, is a major downstream effector molecule of Wnt signaling that regulates the tumor cell cycle as well as proliferation [36]. The luciferase reporter assay results also confirmed that a decrease in LINC00514 expression led to a reduction in TCF/LEF1 activity. To demonstrate the relevance of LINC00514 to Wnt signaling in NSCLC, in vitro experiments using the Wnt signaling activator showed that LiCl significantly reversed the suppressive effect of LINC00514 silencing on cell proliferation, migration, and invasion. These results confirmed that LINC00514 may promote the malignant phenotype of NSCLC by activating Wnt signaling.

EMT is an indispensable process during embryonic development, and beyond its key role in development, it supports various aspects of oncogenesis if it goes unchecked [37]. EMT is a vital factor in tumor cell plasticity and a possible pathway for the generation of drug-resistant cells with mesenchymal properties after epidermal growth factor receptor (EGFR)-targeted therapy [38]. Tumor cells have higher motility and aggressiveness after EMT, in which the reduction of epithelial markers such as E-cadherin and the increase of mesenchymal markers such as N-cadherin are essential hallmarks of this process [39]. The present study confirmed that after LINC00514 knockdown, E-cadherin was upregulated; conversely, N-cadherin was decreased, suggesting that LINC00514 might have a regulatory role in the EMT of NSCLC, and a similar effect has been reported in gastric cancer [40]. Wnt/β-catenin also serves as a common upstream signal for EMT, and an aberrant Wnt signaling in NSCLC, which often induces cellular EMT [41]. Interestingly, emerging investigations have designed some low-toxicity small-molecule drugs targeting less highly conserved regions of the proteins to disrupt protein-protein interactions (PPI), which was effective in reversing EMT in breast cancer cells, showing a surprising inhibition of tumor growth and distant metastasis [42,43]. Hence, disrupting PPI associated with Wnt signaling and EMT in NSCLC may be a promising strategy to explore. In this study, we demonstrated that LIN00514 positively regulates Wnt/β-catenin signaling in NSCLC and promotes EMT. There are limitations in our research, we did not perform gain-of-function by overexpressing LINC00514 to further assess the role of Wnt Signaling and EMT in NSCLC, and the present work has not yet elucidated the specific mechanism by which LINC00514 activates this pathway, which will be the focus of our future investigation.

## Conclusion

Our findings confirmed that LINC00514 is a new oncogene in NSCLC, highlighting that LINC00514 promotes EMT and regulates carcinogenic functions in NSCLC by positively regulating Wnt/β-catenin signaling. Our research demonstrates that LINC00514 holds great promise as a biomarker and effective therapeutic target for patients with NSCLC.

## Data Availability

The datasets generated or analyzed during this study are available from the corresponding author upon reasonable request.
